# Determining a Cut-Off Point for Scores of the Breastfeeding Self-Efficacy Scale–Short Form: Secondary Data Analysis of an Intervention Study in Japan

**DOI:** 10.1371/journal.pone.0129698

**Published:** 2015-06-24

**Authors:** Keiko Nanishi, Joseph Green, Masataka Taguri, Masamine Jimba

**Affiliations:** 1 Department of Community and Global Health, Graduate School of Medicine, The University of Tokyo, Tokyo, Japan; 2 Office of International Academic Affairs, Graduate School of Medicine, The University of Tokyo, Tokyo, Japan; 3 Department of Biostatistics and Epidemiology, Graduate School of Medicine, Yokohama City University, Yokohama, Japan; Centre Hospitalier Universitaire Vaudois, FRANCE

## Abstract

**Background:**

Breastfeeding self-efficacy can be measured with the Breastfeeding Self-Efficacy Scale-Short Form (BSES-SF). Mothers with low BSES-SF scores stop exclusive breastfeeding prematurely, but specific interventions can prevent that undesirable outcome. Because those interventions can be expensive, often one must decide which mothers will receive them. For that purpose, a cut-off BSES-SF score would be useful, but none is available. Therefore, we aimed to assess the overall accuracy of BSES-SF scores as predictors of not practicing post-discharge exclusive breastfeeding, and to choose an appropriate cut-off score for making that prediction.

**Methods:**

This is a secondary data analysis of an intervention study. Data from 378 women in two non-Baby-Friendly Hospitals were analyzed. Participants were women in their third trimester who were 16 years of age or older, were able to read and write Japanese, were expected to have a singleton birth, and had completed the BSES-SF before discharge. BSES-SF scores were measured before discharge. Breastfeeding status was assessed 4 weeks and 12 weeks postpartum. Receiver Operating Characteristic (ROC) curves were used to assess the predictive ability of the BSES-SF and to inform the choice of a cut-off point.

**Results:**

For both of the ROC curves (4 and 12 weeks postpartum) the area under the curve was 0.74. To obtain a high sensitivity, a cut-off score of 50 was chosen. With that cut-off score the sensitivity was 79% and the specificity was 52% 4 weeks postpartum, and they were 77% and 52%, respectively, 12 weeks postpartum.

**Conclusion:**

In conclusion, the BSES-SF has moderate overall accuracy to distinguish women who will not practice exclusive breastfeeding after discharge from those who will. At non-Baby-Friendly hospitals in Japan, interventions to support exclusive breastfeeding might be considered for new mothers who have BSES-SF scores that are less than or equal to 50.

## Introduction

Despite the proven benefits of exclusive breastfeeding [1, 2], many mothers cease it prematurely. In Japan, 96% of mothers intend to breastfeed during pregnancy [3] and almost all women initiate breastfeeding [4]; however, only 52% of mothers were found to predominantly breastfeed their infants 4 weeks postpartum [5].

Breastfeeding self-efficacy can affect breastfeeding initiation, duration, and exclusivity, and it is modifiable [6–10]. Breastfeeding self-efficacy is a mother’s perception of her ability to breastfeed [11], and it is said to be affected by four factors: (1) performance accomplishments (e.g., past breastfeeding experience), (2) vicarious experiences (e.g., watching other women breastfeeding, peer counseling), (3) verbal persuasion (e.g., encouragement from influential others), and (4) physiologic or affective states (e.g., pain, fatigue, anxiety, stress, etc.) [11, 12]. Interventions targeting those four factors can enhance breastfeeding self-efficacy, and also breastfeeding duration and exclusivity [9, 10, 13, 14].

To measure breastfeeding self-efficacy, the Breastfeeding Self-Efficacy Scale-Short Form (BSES-SF) was developed in Canada in 2003 [8]. BSES-SF data are collected via a self-administered questionnaire, which has been translated from English into various languages, including Japanese [15–19]. Results of psychometric tests of the BSES-SF indicate that it can be used in various cultures and with women of various ages.

Mothers with lower BSES-SF scores are more likely to wean their babies from exclusive breastfeeding prematurely than are mothers with higher scores [8, 15, 16, 18, 19]. A longitudinal study with 262 Japanese pregnant women, which included reliability testing and validation testing, indicated that the Japanese version of the BSES-SF could be used to measure breastfeeding self-efficacy. Women were significantly more likely to discontinue exclusive breastfeeding by 4 weeks postpartum if they had a BSES-SF score lower than the sample mean [16]. Therefore, those scores can be used to predict which mothers will stop breastfeeding prematurely, and additional interventions can be offered to those mothers.

For clinical use, a cut-off point among BSES-SF scores would be useful. By using a cut-off point, health professionals can identify mothers who need additional breastfeeding support for exclusive breastfeeding. To the best of our knowledge, no such cut-off point has yet been proposed.

The percentage of mothers who practice exclusive breastfeeding is low, especially in non-Baby-Friendly Hospitals (nBFH) [10]. Thus, we aimed to determine a cut-off point for scores obtained before discharge to identify mothers in nBFHs who were at risk of discontinuing exclusive breastfeeding by 4 weeks and 12 weeks postpartum. We also sought to assess the overall performance of BSES-SF scores as predictors of not practicing exclusive breastfeeding after discharge.

## Materials and Methods

### Study design

This was a secondary analysis of data collected in a self-efficacy intervention study. Those data were collected between August 2010 and January 2011 in Japan [10]. A total of 781 women participated in that intervention study, of whom 574 completed the BSES-SF before discharge. Of those 574 women, 196 were in BFHs and 378 were in nBFHs. For the present study we used data collected from the 378 women at nBFHs. Data from the 196 women at BFHs was not used to avoid influence from the self-efficacy intervention. The intervention improved breastfeeding self-efficacy through 4 weeks postpartum in BFH (*p* = 0.037), however, the positive effect was not observed in nBFHs (*p* = 0.982) [10]. Another reason for the use of data from nBFH was that in Japan the vast majority women deliver in nBFH. While 2,576 facilities provide medical care for delivery in Japan [20], only 75 have been certified as BFH [21]. A cut-off determined using data from nBFH would therefore be generalizable to the majority of women in Japan.

All of the eligible participants were pregnant women in their third trimester who were 16 years of age or older, were able to read and write Japanese, were expected to have a singleton birth, and had completed the BSES-SF before discharge. Exclusion criteria were as follows: (a) an intention to formula-feed or a contraindication to breastfeeding, (b) a pregnancy that ended in either miscarriage or stillbirth, and (c) a medical condition of the women and/or the infant that could significantly interfere with breastfeeding. Further details of the larger study in which these data were collected have been published elsewhere [10].

The current study was approved by the Research Ethics Committee of the Graduate School of Medicine at The University of Tokyo. The data was collected using self-administered questionnaire. Women who read a booklet describing the detail of the study and agreed to participate in the study returned the completed questionnaire. Written consent was not collected in accordance with the Japan’s ethics guideline of epidemiological study. The Research Ethics Committee approved the inclusion of minors without parental consent because: 1) the study was explained in plain language such that it could be easily understood by teenage women, and 2) women often attend the antenatal appointment alone.

### Instrument

We used scores on the Japanese version of the BSES-SF completed before discharge. The BSES-SF comprises 14 items. Each item has 5 response choices on a Likert-type scale, from “not at all confident” (1 point) to “always confident” (5 points). All the items are presented positively and their scores are summed to produce a total score ranging from 14 to 70 [8]. Higher total scores indicate higher levels of breastfeeding self-efficacy.

### Post-discharge exclusive breastfeeding 4 weeks and 12 weeks postpartum

Breastfeeding status was assessed 4 weeks postpartum (by self-administered questionnaire, at the baby’s regular checkup) and again 12 weeks postpartum (by self-administered questionnaire, sent by postal mail with a stamped return envelope). In this study, we were not concerned with breastfeeding practices during hospitalization, so we measured post-discharge exclusive breastfeeding, which we defined as not giving infants any foods or liquids other than breast milk after discharge from the hospital.

### Characteristics of the mothers

To understand the characteristics of the participants, factors known to be associated with breastfeeding were measured during pregnancy or before discharge through self-administered questionnaires. These included age, marital status, economic status, parity, mistimed or unwanted pregnancy, previous experience of breastfeeding, perceived support for breastfeeding from a partner, plan to return to work within 6 months after birth, mode of delivery, infant’s birth weight [16, 22–26]. We also collected data on intentions regarding infant feeding for the first five-to-six months [6, 24], attitude to infant feeding [6, 27] (Iowa Infant Feeding Attitude Scale score [27]), symptoms of depression [23] (Edinburgh Postnatal Depression Scale score [28, 29]), and general family support [22, 24] (Family Apgar score [30]).

### Analysis

Receiver operating characteristic (ROC) curves were used to determine the utility of BSES-SF scores for differentiating between mothers who were at risk to not breastfeed exclusively after discharge from those who were likely to breastfeed exclusively, and to aid in the decision on a cut-off point [31]. An ROC curve is constructed by plotting the true-positive ratio (sensitivity), against the false-positive ratio (1 –specificity). One ROC curve was constructed with the data on post-discharge exclusive breastfeeding 4 weeks postpartum, and one was constructed with data from 12 weeks postpartum. Both were constructed with BSES-SF scores at discharge.

The area under the ROC curve (AUC) was computed because it is an index of the discriminative utility of a test across the full range of possible cut-off points [31]. Higher values of the AUC indicate better discrimination. An AUC of 0.5 indicates a chance result, and the highest possible value of an AUC is 1.0 [32]. Fisher et al. proposed three categories: a test with an AUC greater than 0.9 can be said to be highly accurate, while 0.7–0.9 indicates moderate accuracy and 0.5–0.7 indicates low accuracy [32].

When false-positive and false-negative results are equally undesirable, sensitivity and specificity may be equally weighted, and in such cases Youden’s *J* (*J* = sensitivity + specificity– 1) can be used to identify the optimal cut-off point [33]. *J* is the maximum vertical distance from the ROC curve to the line between (0, 0) and (1, 1). When sensitivity and specificity are equally weighted, the optimal cut-off point is the point with the highest value of *J*. However, when predicting which mothers will not practice post-discharge exclusive breastfeeding, there is no reason to assume that false-negative and false-positive results are equally undesirable, and so there is no reason for sensitivity and specificity to be equally weighted. In such cases one needs to account for “the relative loss (cost) of a false negative as compared with a false positive” [33], that is, the “cost ratio of false-positive and false-negative results” [34].

With the goal of identifying mothers who are not likely to breastfeed exclusively after discharge, we considered a high sensitivity to be more desirable than a high specificity. That is because we considered false-positive results to be more tolerable than false-negative results (i.e., we considered false-positive results to be less costly, less serious, than false-negative results). We arrived at that conclusion by considering the likely consequences of false-negative and false-positive results, as follows. As a consequence of a false-positive result, a health worker would offer an unnecessary intervention, which might result in some unnecessary healthcare expenditure. If resources were very limited, it also might result in the loss of a chance to provide a needed service to a different mother. Nonetheless, a mother who actually does not need the intervention might realize that fact and refuse the unnecessary intervention, or the health worker may eventually notice that it is unnecessary and withdraw it. In contrast, we judged the consequences of a false-negative result to be quite serious. A false-negative result would cause an intervention not to be provided to a mother in need. As a consequence, both the mother and the infant would lose all of the benefits of exclusive breastfeeding.

We could not assign exact numerical values to the consequences of false-positive and false-negative results. However, “the exact cost ratio is not needed in a decision theoretic framework; all that is needed is a qualitative indication of which of the two classification errors is more important to avoid” [35], and we judged that in the present context false negatives are more important to avoid. Therefore, for the cut-off point we decided to choose a BSES-SF score corresponding to a sensitivity higher than the sensitivity at the point with the greatest *J*.

## Results

### Characteristics of participants

Table 1 shows the characteristics of the participants. The majority of them did not have family budget concerns and their education levels were beyond the high-school level. While about half of them had no previous experience of exclusive breastfeeding, the majority of the participants intended to exclusively breastfeed for the first six months after their child’s birth.

**Table 1:** Characteristics of the Participants.

Variable	n (N = 378)	%
**Single mother**	3	0.8
**Having family budget worries**	42	11.1
**Education level of high school or less**		
	91	24.1
**Primiparous**	158	41.8
**Mistimed pregnancy**	89	23.5
**Unwanted pregnancy**	11	2.9
**No experience of exclusive breastfeeding for more than 3 months**		
	209	55.3
**Infant feeding intention for the first six months after birth**		
** Exclusive breastfeeding**	259	68.5
** Partial breastfeeding**	116	30.7
** Not decided**	3	0.8
**Expecting no support for breastfeeding from a partner**		
	119	31.5
**Delivery by caesarian section**		
	50	13.2
**Returning to work within 6 months after delivery**		
	19	5.0
**Iowa infant feeding attitude scale score** ^**a**^		
** Mean (SD)**	62.6	(6.0)
**Family Apgar score** ^**b**^		
** Mean (SD)**	8.4	(2.1)
**Edinburgh Postpartum Depression Scale score** ^**c**^		
** Median (IQR)**	4.0	(1.0–6.0)
**Age (years)**		
** Mean (SD)**	30.8	(4.8)
**Birth weight (g)**		
** Mean (SD)**	3138.0	(327.7)

^a^ Measuring attitude toward infant feeding with 16 items. Total scores range from 16 to 80, with higher scores indicating more positive attitude to breastfeeding.

^b^ Measuring general family support with 5 items. Total scores range from 0 to 10, with higher scores indicating higher levels of family support.

^c^ Measuring depressive symptomatology with 10 items. Total scores range from 0 to 30, with higher scores indicating higher depressive symptomatology.

### Reliability, BSES-SF scores, and post-discharge exclusive breastfeeding

Cronbach’s α of the BSES-SF in this study was 0.94. The mean (SD) of the BSES-SF scores was 42.39 (10.57). The percentages of mothers who reported practicing post-discharge exclusive breastfeeding 4 weeks and 12 weeks postpartum were 15% (48/330) and 9% (29/307), respectively.

### Cut-off score for post-discharge exclusive breastfeeding

**Fig 1:**
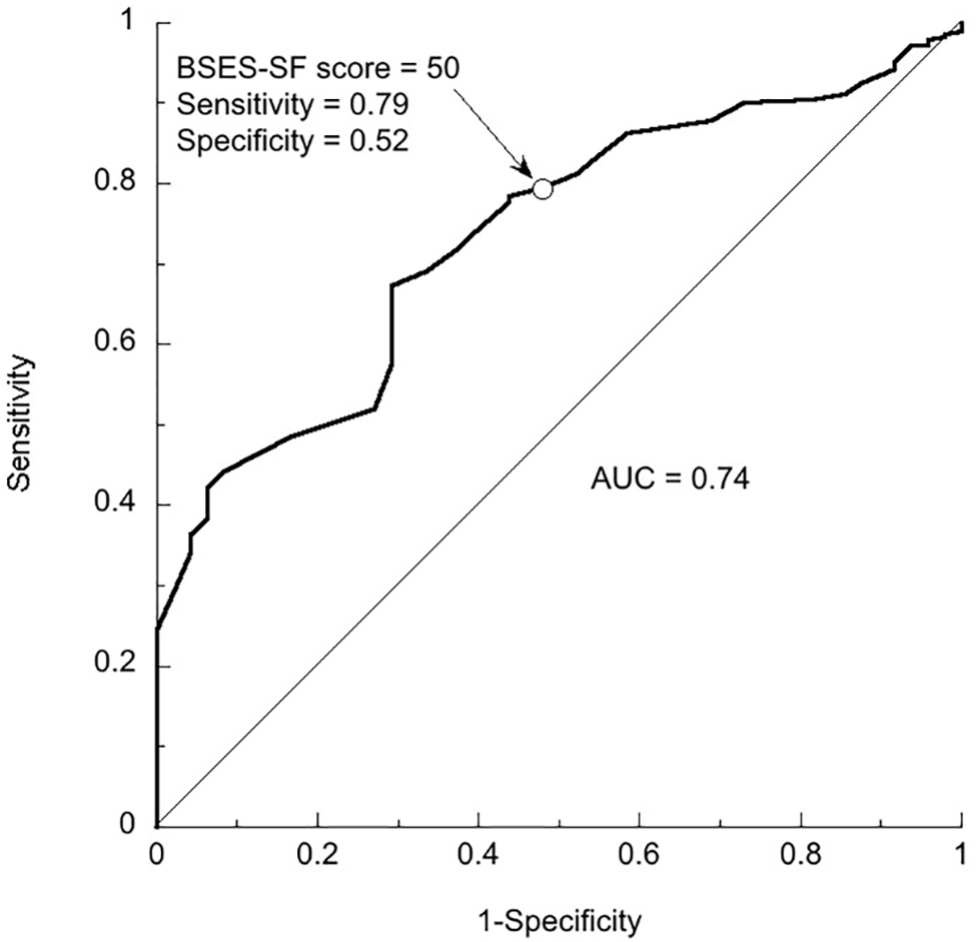
ROC Curve Showing BSES-SF Scores as Predictors of Discontinuation of Exclusive Breastfeeding by the 4th Postpartum Week. The area under the ROC curve is 0.74. The arrow indicates the point corresponding to a BSES-SF score of 50. At that point the sensitivity is 0.79 and the specificity is 0.52.

**Fig 2:**
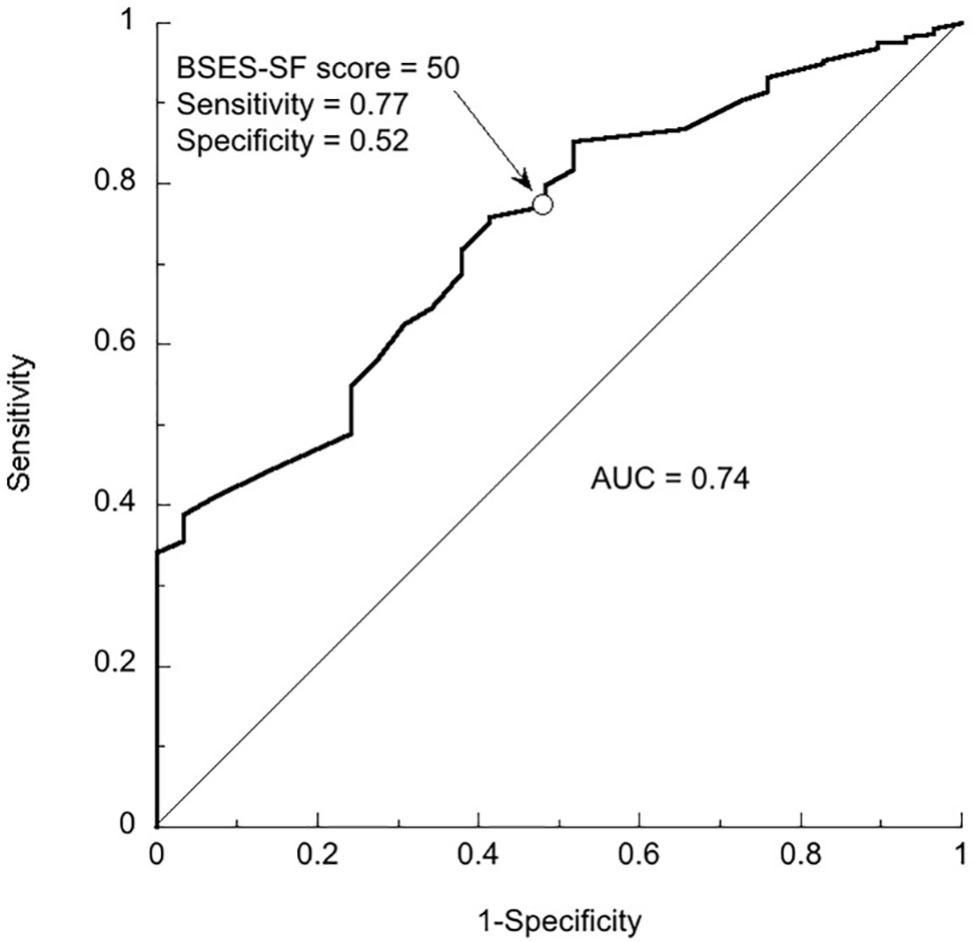
ROC Curve Showing BSES-SF Scores as Predictors of Discontinuation of Exclusive Breastfeeding by the 12th Postpartum Week. The area under the ROC curve is 0.74. The arrow indicates the point corresponding to a BSES-SF score of 50. At that point the sensitivity is 0.77 and the specificity is 0.52.

The ROC curves in Figs 1 and 2 show the accuracy of the BSES-SF scores before discharge as predictors of not practicing post-discharge exclusive breastfeeding at each possible cut-off point. The AUCs were 0.74 (95% CI: 0.70 to 0.80) 4 weeks postpartum and 0.74 (95% CI: 0.66 to 0.82) 12 weeks postpartum. For the two ROC curves, Table 2 shows the coordinates of each point on each curve (that is, sensitivity and 1 –specificity), and the value of *J* at each point. *J* was greatest at BSES-SF scores of 44 (4 weeks postpartum) and 49 (12 weeks postpartum). At a BSES-SF score of 50, the sensitivity and specificity were 79% and 52% (4 weeks), and 77% and 52% (12 weeks). With regard to post-discharge exclusive breastfeeding 4 weeks postpartum in this sample, if the score with the highest value of *J* were used as the cut-off point, the result would be 92 false-negative results. In contrast, if a score of 50 were used as the cut-off point, there would be only 58 false-negative results (Table 3). Given the goal of a high sensitivity (even if that entails low specificity), the cut-off of 50 would be preferred in this context.

**Table 2:** Sensitivity, Specificity and *J* of the BSES-SF at Each Cut-off Points to Predict Exclusive Breastfeeding at 4 Weeks and 12 Weeks Postpartum.

	4 weeks postpartum			12 weeks postpartum		
Score	Sensitivity	1—Specificity	*J*	sensitivity	1—Specificity	*J*
**14**	0.000	0.000	0.000			
**15**						
**16**						
**17**	0.004	0.000	0.004			
**18**						
**19**						
**20**						
**21**	0.007	0.000	0.007	0.000	0.000	0.000
**22**	0.014	0.000	0.014			
**23**	0.018	0.000	0.018	0.004	0.000	0.004
**24**	0.025	0.000	0.025	0.007	0.000	0.007
**25**	0.032	0.000	0.032	0.014	0.000	0.014
**26**	0.043	0.000	0.043	0.025	0.000	0.025
**27**	0.071	0.000	0.071	0.050	0.000	0.050
**28**	0.099	0.000	0.099	0.076	0.000	0.076
**29**	0.135	0.000	0.135	0.108	0.000	0.108
**30**	0.149	0.000	0.149	0.133	0.000	0.133
**31**	0.170	0.000	0.170	0.158	0.000	0.158
**32**	0.213	0.000	0.213	0.209	0.000	0.209
**33**	0.245	0.000	0.245	0.237	0.000	0.237
**34**	0.291	0.021	0.270	0.270	0.000	0.270
**35**	0.340	0.042	0.299	0.309	0.000	0.309
**36**	0.365	0.042	0.324	0.342	0.000	0.342
**37**	0.383	0.063	0.320	0.356	0.034	0.322
**38**	0.422	0.063	0.359	0.388	0.034	0.354
**39**	0.443	0.083	0.360	0.410	0.069	0.341
**40**	0.486	0.167	0.319	0.442	0.138	0.305
**41**	0.521	0.271	0.250	0.489	0.241	0.248
**42**	0.574	0.292	0.283	0.547	0.241	0.305
**43**	0.617	0.292	0.325	0.583	0.276	0.307
**44**	0.674	0.292	0.382	0.626	0.310	0.316
**45**	0.691	0.333	0.358	0.647	0.345	0.303
**46**	0.720	0.375	0.345	0.687	0.379	0.308
**47**	0.741	0.396	0.345	0.719	0.379	0.340
**48**	0.777	0.438	0.339	0.752	0.414	0.338
**49**	0.784	0.438	0.346	0.759	0.414	0.345
**50**	0.794	0.479	0.315	0.773	0.483	0.291
**51**	0.812	0.521	0.291	0.799	0.483	0.316
**52**	0.830	0.542	0.288	0.817	0.517	0.299
**53**	0.862	0.583	0.278	0.853	0.517	0.335
**54**	0.879	0.688	0.192	0.867	0.655	0.212
**55**	0.901	0.729	0.172	0.885	0.690	0.195
**56**	0.904	0.813	0.092	0.903	0.724	0.179
**57**	0.911	0.854	0.057	0.914	0.759	0.155
**58**	0.926	0.875	0.051	0.932	0.759	0.173
**59**	0.943	0.917	0.027	0.950	0.828	0.122
**60**	0.950	0.917	0.034	0.953	0.828	0.126
**61**	0.972	0.938	0.034	0.968	0.897	0.071
**62**	0.972	0.958	0.013	0.971	0.897	0.075
**63**	0.979	0.958	0.020	0.975	0.897	0.078
**64**	0.982	0.979	0.003	0.975	0.931	0.044
**65**	0.986	0.979	0.007	0.982	0.931	0.051
**67**	0.989	1.000	-0.011	0.986	0.966	0.020
**69**	0.993	1.000	-0.007	0.993	0.966	0.027
**70**	1.000	1.000	0.000	1.000	1.000	0.000

**Table 3:** Comparison of Two Possible Cut-off Scores of the BSES-SF: The Score with the Greatest *J* and a Score of 50.

		BSES-SF score with the greatest *J* (4 weeks: 44; 12 weeks:49)	BSES-SF score = 50
**Youden’s *J***			
	**4 weeks**	0.382	0.315
	**12 weeks**	0.345	0.291
**True positives**			
	**4 weeks**	190	224
	**12 weeks**	211	215
**True negatives**			
	**4 weeks**	34	25
	**12 weeks**	17	15
**False positives**			
	**4 weeks**	14	23
	**12 weeks**	12	14
**False negatives**			
	**4 weeks**	92	58
	**12 weeks**	67	63
**Sensitivity**			
	**4 weeks**	67%	79%
	**12 weeks**	76%	77%
**Specificity**			
	**4 weeks**	71%	52%
	**12 weeks**	59%	52%

## Discussion

This was the first attempt to establish a cut-off score on the BSES-SF while assessing the overall performance of BSES-SF scores as predictors of not practicing exclusive breastfeeding after discharge. The results show again that the Japanese version of the BSES-SF can be used to identify women who are likely to stop exclusive breastfeeding prematurely. The AUCs were above 0.7, both 4 weeks and 12 weeks postpartum. Therefore, according to the categories proposed by Fischer et al. [32], the BSES-SF has “moderate” overall accuracy in this context.

Based on the results of this study, we recommend that a BSES-SF score of 50 be used as a cut-off when screening to predict which mothers will not practice post-discharge exclusive breastfeeding, whether 4 weeks or 12 weeks postpartum. Scores less than or equal to 50 can be taken as indicators of a need for interventions to support exclusive breastfeeding. When used as a cut-off, that score correctly identified 79% of the mothers who were not practicing post-discharge exclusive breastfeeding 4 week postpartum, and for the 12-week results the comparable value was 77%.

Any decision regarding a cut-off point involves a trade-off between sensitivity and specificity [36]. For a given test, if one changes the cut-off point to increase sensitivity, then specificity will decrease. As noted above, if the net expected costs of false-negative and false-positive results can be quantified precisely, then the ideal balance between sensitivity and specificity can be computed and the ideal cut-off point can be identified [37]. However, as is the case in almost all such analyses, in this study we could not precisely quantify the total expected costs of false-negative and false-positive BSES-SF results. Nonetheless, “the exact cost ratio is not needed in a decision theoretic framework” [35]. It seems reasonable to consider that for the purposes of this study the cost of a false-negative result would be greater than that of a false-positive result. That led us to favor sensitivity over specificity, and to recommend a score of 50 as the cut-off point. In other settings a different cut-off might be more appropriate, particularly if the word “settings” is interpreted broadly. It should include economic circumstances and practical limitations. For example, if breastfeeding support can be offered at a very low cost, then a cut-off with a higher sensitivity can be used, but if breastfeeding support is very costly, then one might need to use a cut-off that gives less sensitivity and greater specificity.

In this study we used only the BSES-SF total scores. Clinicians can also use the responses to the various BSES-SF questions to design individualized interventions. By checking the responses to each item and understanding what made a particular mother’s score low, a clinician can identify areas in which additional breastfeeding support for that mother should be focused [38].

One limitation of this study is that the longest follow-up was only 12 weeks, while international [39] and national [2, 40] organizations recommend exclusive breastfeeding for the first 6 months of life. We cannot be sure how accurate the BSES-SF scores would be as predictors of events beyond 12 weeks postpartum. Another limitation stems from the fact that the data we used came from a self-efficacy intervention study. That intervention did not have a statistically significant effect on breastfeeding self-efficacy or exclusivity [10], but replication studies in other groups would be helpful. Finally, we note again that the participants were recruited through convenience sampling at two nBFHs in Japan, and that the majority were married, educated, and not in financial distress. Further study is needed of the relevance of the recommended cut-off point to other groups such as socioeconomically deprived women.

## Conclusions

This is, to the best of our knowledge, the first study to establish a cut-off point for BSES-SF scores in Japan. That cut-off point can be used to identify mothers who are likely to stop exclusive breastfeeding prematurely. We can recommend that, at nBFH in Japan, interventions to support exclusive breastfeeding should be considered for new mothers whose scores on the Japanese version of the BSES-SF are less than or equal to 50.

Additional study is warranted to find out whether BSES-SF scores can be used to predict exclusive breastfeeding 6 months postpartum, and also to answer questions about generalizability to other groups.
